# Coupled mutation finder: A new entropy-based method quantifying phylogenetic noise for the detection of compensatory mutations

**DOI:** 10.1186/1471-2105-13-225

**Published:** 2012-09-11

**Authors:** Mehmet Gültas, Martin Haubrock, Nesrin Tüysüz, Stephan Waack

**Affiliations:** 1 Institute of Computer Science, University of Göttingen, Goldschmidtstr. 7, Göttingen, 37077, Germany; 2 Department of Bioinformatics, University of Göttingen, Goldschmidtstr. 1, 37077 Göttingen, Germany; 3 Erasmus MC Stem Cell Institute, Department of Cell Biology, Erasmus Medical Center, Rotterdam, The Netherlands

## Abstract

**Background:**

The detection of significant compensatory mutation signals in multiple sequence alignments (MSAs) is often complicated by noise. A challenging problem in bioinformatics is remains the separation of significant signals between two or more non-conserved residue sites from the phylogenetic noise and unrelated pair signals. Determination of these non-conserved residue sites is as important as the recognition of strictly conserved positions for understanding of the structural basis of protein functions and identification of functionally important residue regions. In this study, we developed a new method, the Coupled Mutation Finder (*CMF*) quantifying the phylogenetic noise for the detection of compensatory mutations.

**Results:**

To demonstrate the effectiveness of this method, we analyzed essential sites of two human proteins: epidermal growth factor receptor (EGFR) and glucokinase (GCK). Our results suggest that the *CMF* is able to separate significant compensatory mutation signals from the phylogenetic noise and unrelated pair signals. The vast majority of compensatory mutation sites found by the *CMF* are related to essential sites of both proteins and they are likely to affect protein stability or functionality.

**Conclusions:**

The *CMF* is a new method, which includes an MSA-specific statistical model based on multiple testing procedures that quantify the error made in terms of the false discovery rate and a novel entropy-based metric to upscale BLOSUM62 dissimilar compensatory mutations. Therefore, it is a helpful tool to predict and investigate compensatory mutation sites of structural or functional importance in proteins. We suggest that the *CMF* could be used as a novel automated function prediction tool that is required for a better understanding of the structural basis of proteins. The *CMF* server is freely accessible at http://cmf.bioinf.med.uni-goettingen.de.

## Background

A multiple sequence alignment (MSA) of proteins contains a set of aligned amino acid sequences in which homologous residues of different sequences are placed in same columns. Therefore, functionally or structurally important amino acids and their positions both of which are often strictly conserved are easily detectable with MSAs [1-3]. On the other hand, detection of important non-conserved residue positions related to several essential conserved residues requires a more sophisticated approach. The usage of methods such as correlation analysis allow the identification of important non-conserved residue positions based on their correlated mutation manners [4,5] due to functional coupling of mutation positions. This coupling might stem from one mutation in a certain site affecting a compensating mutation at another site, even if both related residue sites are distantly positioned in the protein structure. Moreover, these coupled mutations can result from spatial, physical, or chemical restrictions or signaling of allostery [4,5]. Thus, determination of these positions is as crucial as the recognition of strictly conserved positions for the understanding of the structural basis of protein functions, and for the identification of functionally important residue regions which might be disease associated, responsible for the maintenance of internal protein volume, or possibly form key sites for interactions within or between proteins [6-9].

Until now, a variety of studies have employed Pearson’s correlation coefficient methods [10-12], perturbation based methods [9,13] and mutual information (MI) based methods [6,14-17] because of their simplicity and efficiency for the detection of coupled mutations in MSAs. However, due to background noise, all of these methods interfere with the identification of compensatory mutation signals [14,18,19]. Hence, the significant compensatory mutation signals must be separated from the background noise that might occur as a result of: i) false signals arising from insufficient data; ii) sites with low or high conservation biasing the signal; iii) phylogenetic noise. While the first two types of noise can be easily overcome by appropriately filtering the data [16], phylogenetic noise can only be eliminated to some extent by excluding highly similar sequences from the MSA [19].

Recently, several methods such as bootstrapping, simulation or randomization methods have been utilized in order to minimize the influence of phylogenetic linkage and stochastic noise [15,20,21]. Dunn et al. [19] have introduced the *average product correction* (APC), to adjust MI for background effects. Merkl and Zwick, in their study, [16] have used a normalized MI (see Equation 1) and focused on only 75 residue pairs with the highest normalized MI values as significant for each MSA. Gao et al. [17] have pursued a similar approach, where they have replaced the metric used in [16] with the amino acid background distribution (MIB). While the reduction of background noise in the model of Dunn et al. is not quantified, the approaches of Gao et al. and Merkl and Zwick appear to be over-conservative, yet specific.

Despite the presence of a variety of different methods as mentioned above, to date there is still need for a method to deal with the noise as well as for powerful metrics to improve the sensitivity. In this study, we have developed such a method called Coupled Mutation Finder (CMF). The CMF includes an MSA-specific statistical model based on multiple testing procedures described in [22,23] and a novel entropy-based metric to upscale dissimilar compensatory mutations and also to complement the normalized MI metric used in [16]. Unlike previous normalized MI based studies [16,17], we have separated metric-based significant compensatory mutation signals from background noise with respect to our MSA specific statistical model that quantifies the error made in terms of the false discovery rate.

To demonstrate the performance and functionality of the CMF, we analyzed the structurally or functionally important positions of two human proteins, namely epidermal growth factor receptor (EGFR) and glucokinase (GCK). The proteins have been chosen because their functionally and structurally important positions have been experimentally well studied previously [24-35]. As a result, the *CMF* detects in these two proteins disease associated amino acid mutations (non-synonymous single nucleotide polymorphisms (nsSNPs)), not strictly conserved catalytic or binding sites, and residues that are nearby one of these sites or in the close neighborhood of a strictly conserved positions, which are likely to affect protein stability or functionality [36-38].

## Results

Our method to predict functionally or structurally important residue positions is composed of two steps. First, we have devised a new MSA-specific statistical method for the identification of significant MSA column pairs with respect to either of the two metrics U and $U_{D(\alpha )}$. Assume that *M* is the MSA under study, these pairs are annotated as (U,M)-significant and $\left(U_{D(\alpha )},M\right)$-significant, respectively. Second, we utilized the connectivity degree of a residue site with respect to a metric introduced in [16]. The connectivity degree of a residue site indicates the number of its significant coupled mutation pairs. In this case, a site is called (U,M)-significant when the frequency of occurrence of this site in the set of (U,M)-significant pairs exceeds the 90-th percentile. Having defined the concept of a $\left(U_{D(\alpha )},M\right)$-significant site analogously, a site is defined as *CMF*-significant with respect to *M*, when it is either (U,M)-significant or $\left(U_{D(\alpha )},M\right)$-significant.

In this study, we analyzed human EGFR (pdb entry 2J6M) and GCK (pdb entry 1V4S) proteins with a false discovery rate (*FDR*) of 1%. For the preceding one, we defined a total of 14339 out of 26079 non-conserved column pairs as significant. 11365 of these significant pairs are detected as (U,M)-significant and 3798 pairs are observed as $\left(U_{D(\alpha )},M\right)$-significant. Only 824 EGFR pairs are significant with respect to both metrics. On the other hand, for GCK, we identified a total of 32654 out of 69645 non-conserved column pairs as significant where 18106 of them are U-significant and 16241 are $U_{D(1)}$-significant. Moreover, 1693 pairs are defined as significant for both U and $U_{D(1)}$-significant.

Applying the connectivity degree technique, we identified 22 and 36 residue positions as U-significant for human EGFR and GCK proteins, respectively. Additionally, 21 positions of EGFR and 36 positions of GCK were detected as $U_{D(1)}$-significant. Finally, a total of 43 sites of EGFR and 72 of GCK were found as *CMF*-significant, and predicted as of structural or functional importance. However, there have been no residue sites defined as significant with respect to either metric.

### Essential sites of human EGFR and GCK proteins

To evaluate the *CMF*-significant residue sites, we have investigated essential sites of human EGFR (pdb entry 2J6M) and GCK (pdb entry 1V4S) proteins. The essential sites of both proteins have been assigned into three main categories: i) the nsSNP positions and their adjacent sites; ii) residue positions which are located at or near active sites, allosteric sites, or binding sites; iii) residue positions which are nearby strictly conserved sites. Here, we have used “nearby” definition of Nussinov et al. [39] and defined two residues as in contact or adjacent when the distance between their major carbon atoms is less than 6 Å. We have defined positions which are nearby nsSNPs as essential, because several of them are also adjacent to active sites, allosteric sites, binding sites, or strictly conserved sites. Thus, we refer to a *CMF*-significant residue site as “functionally or structurally important” if it falls into one of these categories of essential sites.

### Position analysis of the Human Epidermal Growth Factor Receptor (EGFR) protein

**Figure 1:**
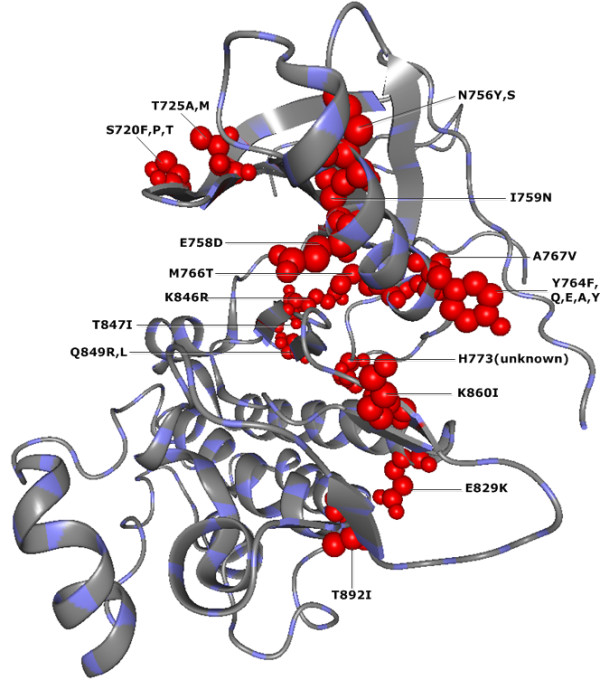
***CMF*-significant nsSNP positions in human EGFR protein (PDB-Entry 2J6M).** The red spheres correspond to structural localization of 15 different nsSNP positions found by CMF as significant in the EGFR protein.

The epidermal growth factor receptor (EGFR) is a member of the ErbB (Erythroblastic Leukemia Viral Oncogene Homolog) family receptors. Signaling through this receptor is a highly conserved mechanism from nematode to humans involved in numerous processes, including proliferation, cell fate determination, and tissue specification [40]. Furthermore, several studies have implicated that mutations resulting in misregulation of the activity or action of EGFR led to multiple cancers, including those of the brain, lung, mammary gland, and ovary [24-27]. Here, in order to detect essential mutation positions in corresponding sequence of human EGFR protein, we determined altogether 43 *CMF*-significant residue sites (see Additional file 1). 15 of these significant residue sites have been verified as nsSNP sites through the Ensembl database annotation and they are illustrated in Figure 1.

**Figure 2:**
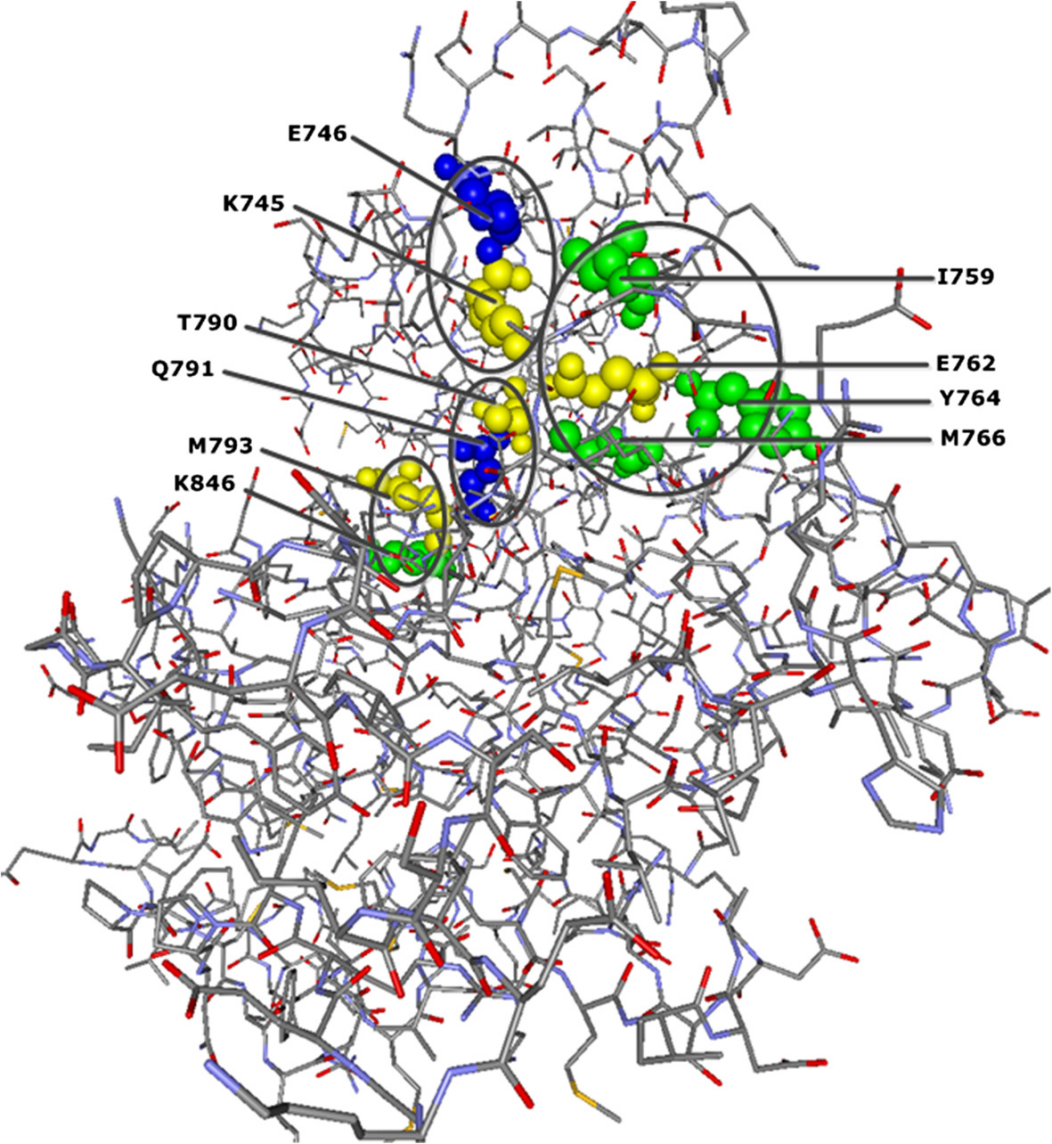
***CMF*-significant residue positions are in contact with gefitinib binding sites in human EGFR protein (PDB-Entry 2J6M).** Yellow spheres denote positions of the gefitinib binding sites in the wild type kinase. Blue spheres show the localization of significant adjacent residue positions found by CMF which are in contact with these binding sites. Moreover, the CMF-significant sites I759, Y764, M766 and K846, shown with green spheres, are already described as nsSNP positions and they are also in contact with gefitinib binding sites E762 and M793, respectively. The circles indicate clusters of gefitinib binding sites and their significant adjacent sites.

Additionally, the significant sites E746, Q791, and four of the nsSNP positions (I759,Y764,M766 and K846) are also in contact with critical active site regions for gefitinib binding site in the wild type EGFR kinase [25,28] (see Figure 2).

Moreover, we observed that 17 further CMF-significant positions are essential sites (see Table 1). In total, we have established here for EGFR protein the importance of 34 out of 43 CMF-significant residue sites via different resources [25,28,35].

**Table 1:** *CMF*-significant essential sites in human EGFR protein, which are nearby either nsSNPs or strictly conserved sites

***CMF*-significantessential sites**	**Nearby nsSNPs, or strictlyconserved sites**	**Reference**
Y727	726^**c**^ 743^**c**^	-
*H*755	756^**s**^, 758^**s**^	[35]
*D*800	798^**c**^	-
*G*824	773^**s**^	[35]
*D*830	829^**s**^	[35]
*E*868	892^**s**^	[35]
*E*872	873^**s**^	[34]
*V*876	877^**c**^	-
*K*879	877^**c**^, 880^**c**^	-
*Y*891	892^**s**^, 895^**c**^	[35]
*S*899	880^**c**^, 896^**c**^, 898^**c**^, 901^**c**^	-
*Y*900	898^**c**^, 901^**c**^	-
*T*909	906^**c**^, 936^**c**^	-
*S*912	906^**c**^, 936^**c**^	-
*K*913	914^**c**^	-
*D*916	914^**c**^	-
*M*947	901^**c**^, 950^**c**^	-

^**s**^: non-synonymous snp site,^**c**^: strictly conserved site.

Although the vast majority of* CMF*-significant sites are verified to be structurally or functionally important in human EGFR protein, nine* CMF*-significant sites do not overlap with essential sites. The reason for the high connectivity degree of these unconfirmed significant sites and their role in the EGFR protein is unclear.

### Position analysis of the Human Glucokinase (GCK) protein

Glucokinase (GCK) is a monomeric enzyme catalyzing phosphorylation of glucose to glucose-6-phosphate, which is the first step in the utilization of glucose, at physiological glucose concentration in pancreas and liver. Given the fact that GCK displays low affinity for glucose, it acts as a glucose sensor playing an important role in the regulation of carbohydrate metabolism. Mutations of the GCK gene can lead to maturity onset diabetes of the young (MODY) characterized by an autosomal dominant mode of inheritance and onset early adulthood [32], or familial hyperinsulinemic hypoglycemia type 3 (HHF), common cause of persistent hypoglycemia in infancy [41].

**Figure 3:**
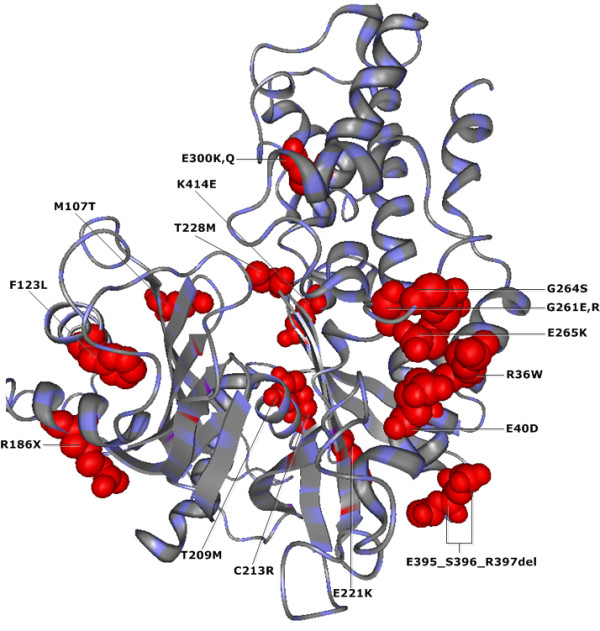
***CMF*-significant nsSNP positions in human GCK protein (PDB-Entry 1V4S).** Red spheres show the structural localization of 16 different nsSNP positions found by*CMF* as significant in the GCK protein.

Applying our method, we found 72 CMF-significant sites to be structurally or functionally important in human GCK protein (see Additional file 2). 16 of these significant residue positions are related to disease associated nsSNP positions [29-31,34,35] (see Figure 3).

**Figure 4:**
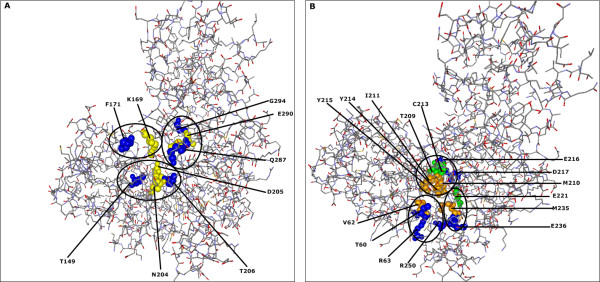
***CMF*-significant residue positions are in contact with glucose binding site and allosteric site in human GCK protein (PDB-Entry 1V4S).** (**A**) Yellow spheres show the structural positions of the glucose binding sites (active sites). Blue spheres correspond to localization of significant adjacent residue positions found by* CMF* which are in contact with these active sites. (**B**) Orange spheres denote the allosteric sites. Blue spheres correspond to localization of just significant adjacent residue positions and green spheres indicate the significant residue positions which are already described as nsSNP position and in contact with these allosteric site. Additionally, the significant position R63 is allosteric site by itself and it is also in contact with an other allosteric site. The circles indicate clusters of glucose binding sites (**A**), allosteric sites (**B**), and their significant adjacent sites.

Furthermore, nine significant sites are found to be in contact with allosteric sites in the GCK protein structure. Among these sites, the R63 is also allosteric site by itself [32] and T209, C213 and E221 overlap with nsSNP regions (see Figure 4B). Moreover, the five significant sites T149, F171, T206, Q287, and G294 interact with glucose binding sites K169, D204, N205, and E290 [32] (see Figure 4A).

Besides this, there are further 30* CMF*-significant essential sites which are nearby nsSNPs or strictly conserved residue positions (see Table 2). Altogether, we showed the functionality of 57 positions out of 72 CMF-significant residue sites via different resources [29-35].

**Table 2:** *CMF*-significant essential sites in human GCK protein, which are nearby either nsSNPs or strictly conserved sites

***CMF*-significant essential sites**	**Nearby nsSNPs or strictly conserved sites**	**Reference**
M34	36^***s***^	[34]
*T*65	66^**c**^	
*E*67	66^**c**^, 68^**c**^	-
*T*82	81^**c**^	-
*N*83	81^**c**^, 108^**s**^, 110^**s**^	[34]
*H*105	106^**s**^	[29]
*C*129	131^**s**^, 132^**s**^	[29,34]
*F*133	131^**s**^, 132^**s**^	[29,34]
*F*148	147^**c**^, 150^**c,s**^	[34]
*F*152	150^**c,s**^, 151^**c**^	[34]
*H*156	162^**s**^	[29]
*N*180	162^**s**^, 182^**s**^	[29,34]
*F*260	257^**s**^, 258^**c**^, 259^**s**^, 261^**s**^	[34]
*D*262	259^**s**^, 261^**s**^, 264^**s**^	[34,42]
*L*266	261^**s**^, 264^**s**^, 265^**s**^	[29,34,42]
*D*267	264^**s**^, 265^**s**^	[29,42]
*L*271	274^**c**^	-
*S*281	278^**c**^, 279^**s**^	[34]
*Q*286	259^**s**^	[34]
*E*331	299^**c,s**^	[34]
*T*332	295^**c**^, 299^**c,s**^	[34]
*R*333	336^**s**^	[34]
*Q*337	336^**s**^	[34]
*E*339	336^**s**^	[34]
*N*391	392^**s**^	[29,34]
*S*411	227^**c,s**^, 410^**c**^, 414^**s**^	[34]
*S*418	416^**s**^	[30]
*F*419	416^**s**^	[30]
*E*442	444^**c**^	-
*E*443	444^**c**^, 445^**c**^	-

^**s**^: non-synonymous snp site,^**c**^: strictly conserved site.

While we are able to establish the large number of*CMF*-significant sites as structurally or functionally important in human GCK protein, 15*CMF*-significant sites do not overlap with essential sites. Their importance in the GCK protein and the reason of high connectivity degree of these unconfirmed significant sites has not been explicitly determined yet.

### A comparison between U-metric and $U_{D(\alpha )}$-metric

Similarities in physical or biochemical properties of amino acids are likely to be crucial for the detection of functionally or structurally important positions of a protein. In contrast to the U-metric, which is a normalized mutual information that uses only the frequencies of occurrences of amino acids in the MSA columns, the novel $U_{D(\alpha )}$-metric includes dissimilarities according to the BLOSUM62 matrix when calculating normalized mutual information. As a result the positions which have undergone dissimilar compensatory mutations are upscaled.

Having applied the U-metric as well as the $U_{D(\alpha )}$-metric to human EGFR and GCK proteins, the $U_{D(\alpha )}$-metric has shown better sensitivity and specificity. However, only when we use the both metrics together, the sensitivity is significantly increased, whereas the specificity is only moderately decreased. The details are presented in Table 3.

**Table 3:** **Comparison between**U**-metric and**$U_{D(\alpha )}$**-metric**

	**Sensitivity**	**Specificity**
U-significance	9.7%	91.5%
$U_{D(\alpha )}$-significance	12.4%	97.2%
*CMF*-significance	22.1%	88.7%

It is important to note that the two metrics complement each other. Thus, we propose to use them together.

### ***CMF*** as a Web service

We have implemented a* CMF* Web service ( http://cmf.bioinf.med.uni-goettingen.de) that takes an MSA in multiple FASTA format and a real number from (0,1) interpreted as false discovery rate as input. It reports the results via email.

## Discussion

To predict sites of structural or functional importance, we combine the known U-metric of normalized mutual information [16] with a novel metric $U_{D^{\left(i\right)}(1)}$ to enhance the influence of dissimilar compensatory mutations when measuring covariation of two sites. We discuss how we devised $U_{D(1)}$ in Methods section.

To learn the frequency of compensatory mutations, we took U-significant site pairs as training data. We did that for reasons of computation time regardless of the fact that these data are biased. To deal with this bias, one could carry through the training in an iterative process, with our training being the first iteration. For *i *> 0, in the (*i* + 1)-th iteration of this modified training, a doubly stochastic matrix $\left(D_{\text{CompMut}}^{\left(i+1\right)}\right)$ is calculated based on $U_{D^{\left(i\right)}(1)}$-significant site pairs. This is done until the training data are stable.

According to Birkhoff’s Theorem [43], every doubly stochastic matrix is a convex combination of permutation matrices. Moreover, from the Hardy-Littlewood-Pólya majorization theorem [44] follows that transforming the probability mass function by a doubly stochastic matrix increases entropy. Consequently, by linearly transforming the empirical amino acid pair distribution of a site pair by *D*(1) before calculating the U-value, we penalized those site pairs whose original distribution does not match the frequency pattern of formal dissimilar compensatory mutations in the training data described in the Methods section.

The challenge was to separate the signal caused by structural and functional constraints from the background. To address this issue, we studied only metrics *μ* that satisfy the following condition. The larger the *μ*(*k*,*l*)-value, the larger the probability that the two sites* k* and* l* have co-evolved. Our critical assumptions were: i) the* μ*(*k*,*l*)-values follow three different distributions, one for the signal, one for the noise, and one for pairs of completely unrelated sites; ii) there is an MSA-dependent threshold below which the metric *μ* does not fall with overwhelming probability, when it is applied to the site pairs of functional or structural importance to which* μ* is sensitive; iii) there is an MSA-dependent threshold significantly smaller then the one in (ii) such that with overwhelming probability there are no* μ*(*k*,*l*)-values of pairs (*k,l*) of unrelated sites exceeding it.

In order to near-completely eliminate the noise, we filtered both our training and input data. We calculated the significant pairs such that the preassigned false discovery rate was guaranteed by generalizing the Storey-Tibshirani procedure devised for multiple testing problems [22].

Our method to eliminate noise is orthogonal to the technique developed in [19]. Therein, for every pair of sites the so-called average product correction (APC) is calculated as an explicit noise measure, by which the mutual information is then decreased. Furthermore, it generalizes the way Merkl and Zwick [16] as well as Gao et al. [17] cope with noise. According to our judgment, taking only the top 75 high-scoring pairs or the top 25 pairs into account as done in [16,17], respectively, is too conservative.

We based our noise separation technique on rather weak distribution assumptions that are standard practice in multiple hypothesis testing, instead of explicitly model the noise in terms of a metric. We applied the connectivity degree technique due to Merkl and Zwick [16] to significant site pairs with respect to our metrics. The cut-off for the connectivity degree was set to the 90-th percentile. That way we defined significant sites. Finally, a site was defined to be* CMF*-significant, if it was* μ*-significant, where* μ* is either U or $U_{D(1)}$.

Why did we set the cut-off value for the connectivity degree to the 90-th percentile? Going through all possible*n*-th percentiles for* n *= 80,81,…,99, the Matthews correlation coefficient (MCC) of a joint prediction for human EGFR and GCK proteins is maximal if* n *= 90.

It is plausible that the number of functionally or structurally important sites does not only depend on the length of the protein. Therefore, the 90-th percentile cut-off should be replaced by an MSA-dependent threshold in future studies.

Our results for human EGFR and GCK proteins suggest that the large majority of significant compensatory mutation sites found by* CMF* are in agreement with previous experimental studies regarding the functions and stability of these proteins. 15 and 16* CMF*-significant sites in human EGRF and GCK proteins, respectively, are verified as disease associated nsSNP positions (see Figures 1 and 2) where most amino acid substitutions in protein sequences damage structural stability of proteins [36,37,45]. Moreover, we have observed that in both proteins some of* CMF*-significant nsSNP positions are nearby allosteric sites, binding sites or catalytic sites each of which are considered to be functionally important [46,47] (see Figures 2 and 4). Disease associated mutations at these nearby positions are likely to affect protein function [38,48].

Despite the large number of* CMF*-significant sites demonstrated to be structurally or functionally important for both of the proteins, 9 and 15 significant sites in human EGFR and GCK proteins, respectively, are not included in essential sites. However, we hypothesize that most of the novel significant sites may play a critical role in both proteins notwithstanding the absence of previous experimental data. Therefore, further progress from the molecular and structural biology end is required not only to assess the importance of these sites, but also for a future perspective on a deeper understanding of protein structure.

Because we have also used the U-metric, we compared our tool with H2r presented in [16] rather than with those methods developed in [17]. This way, we studied the impact of applying the Storey-Tibshirani procedure in combination with the effect of using the 90-th percentile cut-off for the connectivity degree. We have applied H2r without adding pseudo counts to the human EGFR and GCK protein. For EGFR, the 14 sites T725, A755, N756, A767, Q791, V802, N816, V819, K846, V876, M881, K913, D916, and E931 are identified as significant. Out of these significant sites, ten of these residue sites T725, A755, N756, A767, Q791,K846, V876, M881, K913, and D916 are essential sites. On the other hand, for GCK, H2r identified the 15 residue positions L25, R36, R63, M107, C213, V226, G261, D262, G264, L266, D267, E268, T405, K414, and H416 as significant. Twelve of these sites, namely R36, R63, M107, C213, V226, G261,D262, G264, L266, D267, K414, and H416, are essential sites. However, when using the H2r Web service ( http://www-bioinf.uni-regensburg.de/) to analyze EGFR and GCK proteins, sensitivity is decreased, while precision is increased. By this service only eight sites for EGFR and nine sites for GCK were found to be significant. Moreover, only five and eight of them are verified as functionally or structurally important for EGFR and GCK proteins, respectively. This difference stems from the fact that the H2r Web service tightens the filtering of the columns. In addition to this, statistically evaluating H2r for EGFR and GCK proteins, we observed a sensitivity of 5.4%, specificity of 96.7%, precision of 75.9%, and a Matthews correlation coefficient value of 0.047. On the other hand, the CMF reaches precision of 79.1%, and a Matthews correlation coefficiant value of 0.133. For sensitivity and specificity of the* CMF* refer to the last row of Table 3.

The results of this comparison show that a vast majority of functionally or structurally important residue positions cannot be detected without using our novel MSA specific model and both metrics ( U and $U_{D(1)}$) together.

## Conclusions

The* CMF* is a new method which includes an MSA-specific statistical model based on multiple testing procedures that quantifies the error made in terms of the false discovery rate and a novel entropy-based metric to upscale BLOSUM62 dissimilar compensatory mutations. Hence, it shows how dissimilar compensatory mutations have affected genomic sequences in the course of evolution. The method is able to predict significant compensatory mutation positions in protein sequences. We suggest that CMF could be used as a novel automated function prediction tool that is required for a better understanding of the structural basis of proteins.

## Methods

**Figure 5:**
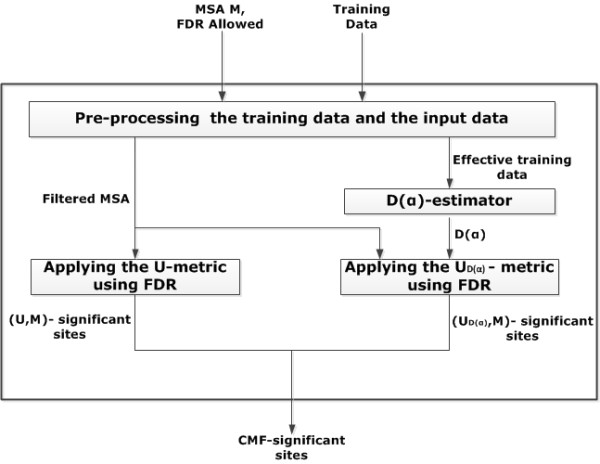
**Flowchart of the *CMF*-analysis.**

In this section we describe the training data used and the methods applied and partly developed. Our descriptions follows the structure of Figure 5, i.e. we start with the data and the preprocessing and systemically work towards the* CMF*-significant site prediction.

### Training data set and pre-processing

We used a redundancy free set of more than 35000 protein structures computed in Rainer Merkl’s Lab at the University of Regensburg in the following way. The protein structures were taken from the protein data base ( http://www.pdb.org/). The PISCES services [49] was applied to assess proteins on sequence similarity and equality of 3D-data. The related MSAs were gathered from the HSSP data base ( http://swift.cmbi.ru.nl/gv/hssp/).

Taking pattern from [16], we filtered every MSA obtained as follows. First, highly similar and dissimilar sequences were deleted to ensure that the sequence identity between any two sequences is at least 20% and no more than 90%. Second, we removed strictly conserved residue columns, where the percentage of identical residues is greater than 95%. Third, we eliminated the residue columns which contain more than 25% gaps. Finally, we discarded all MSAs with less than 125 sequences. More than 17000 MSAs survived the last filtering step. We used approximately 1700 MSAs as training data which we randomly chose from this set. The pdb entries of the corresponding protein structures are listed in Additional file 3.

### Detecting compensatory mutations by the U-metric

In [16] a normalized measure of mutual information ranging over the interval [0,1] is successfully used to detect important residues. It is defined as

(1) $$U\left(i,j\right):=2\cdot \frac{H\left(i\right)+H\left(j\right)-H\left(i,j\right)}{H\left(i\right)+H\left(j\right)},$$

where $H\left(i\right)$ and $H\left(j\right)$ are the entropy of the empirical amino acid distributions of the columns* i* and* j*, and $H\left(i,j\right)$ is their joint entropy.

We determine an MSA-dependent threshold* τ *above which U-values are defined as significant. Let* M* be the MSA for the protein under investigation. We extend a standard approach of multiple testing theory [22,50,51] with the following assumptions in mind.* M*’s $U\left(k,l\right)$-values follow three different distributions. The null distribution* F*_0_ represents background signals. The distributions* G*_1 _and* G*_2 _model the unrelated pairs and the signal pairs, respectively.

We assume* F*_0_ to be a* β *-distribution, and* M*’s $U\left(k,l\right)$-values* U*_1_,*U*_2_,…,*U*_*μ*_ to be an independent and identically distributed (iid) sample.

**Figure 6:**
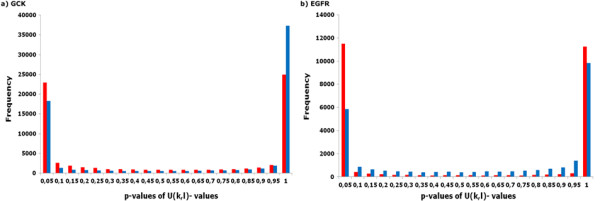
**Four *p*-value distributions of (transformed) normalized mutual information values for human GCK and EFGR proteins having PDB-ID 1V4S and 2J6M, respectively.** The bar charts illustrate the two steps of our model: i) blue bars show the* p*-value distribution of the $U\left(i,j\right)$-scores; ii) red bars display the* p*-value distribution of the $U_{D(1)}\left(i,j\right)$-values. The p-values close to zero represent the significant pairs by means of which we assess the individual residue position. As one can see, within [0.25,0.70] these four distributions are approximately uniform.

Let* X*_*ι*_:= 1−*F*_0_(*U*_*ι*_) be the*p*-value of* U*_*ι*_ with respect to*F*_0_. If* U*_*ι*_ is *F*_0_-distributed, then* X*_*ι*_ is uniform over [0,1]. However, if* U*_*ι*_ is* G*_1_-distributed or* G*_2_-distributed, then* X*_*ι *_is skewed to 1 or to 0 (see Figure 6). According to [22,23], the fraction* γ* of the* U*_*ι*_’s that are *F*_0_-distributed is estimated by

$$\hat{\gamma }:=\frac{\text{number of}\;p\text{-values in}[\lambda_{1},\lambda_{2}]}{\mu (\lambda_{2}-\lambda_{1})}.$$

The tuning parameters* λ*_1 _and* λ*_2 _are chosen such that the fraction of not uniformly distributed* p*-values that fall into [*λ*_1_,*λ*_2_] is negligible.

We call a pair of sites (*i,j*) of the protein under study ( U,M)*-significant* if and only if the*p*-value $1-F_{0}(U\left(i,j\right))$ is less than or equal to* τ*, for a threshold* τ *≤* λ*_*1 *_that ensures the input false discovery rate* FDR*, which in turn can be estimated by

$$\hat{\text{FDR}}(\tau )=\frac{\hat{\gamma }\mu \tau }{\text{number of}\;p\text{-values}\leq \tau }.$$

In order to determine the parameters of the* β*-distribution* F*_0_, it is sufficient to estimate the expected value and the variance. The expected value is estimated by the sample mean of all U -values of* M*. As for the variance, we take pattern from [52]. Having drawn an iid sample $\left(\left(C_{1},C_{1}^{\prime }),(C_{2},C_{2}^{\prime }),\ldots ,(C_{\nu },C_{\nu }^{\prime }\right)\right)$ of random column pairs of a sufficient size whose U-values fall in a preassigned subinterval of [0,1], we calculate* D*_1_,*D*_2_*,…,D*_*ν*_ by randomly shuffling $\left(C_{\iota }^{\prime }\right)$ for every* ι *=1,2,…,*ν*. The variance is then estimated as the sample variance of (*C*_1_,*D*_1_),(*C*_2_,*D*_2_),…,(*C*_*ν*_,*D*_*ν*_).

The* connectivity degree* of a site* i* with respect to the metric U and the MSA*M* is defined as number of sites*j* such that (*i*,*j*) is (U,M)-significant [16]. Site* i* is defined to be (U,M)*-significant*, if* i*’s connectivity degree with respect to U and* M* is greater than or equal to the 90-th percentile. The (U,M)-significant sites of a protein do not coincide with those predicted by H2r [16]. The connectivity degrees attained and the threshold used substantially differ. In particular, the latter one is data-dependent rather than constant.

### Enhancing prediction by the $U_{D(\alpha )}$-metric that models dissimilar compensatory mutations

A pair ((*a*_*i*_*,a*_*j*_),(*a*_*k*_,*a*_*l*_)) of amino acid pairs is defined to be a* formal dissimilar compensatory mutation*, if the BLOSUM62 score both of (*a*_*i*_*,a*_*k*_) and (*a*_*j*_*,a*_*l*_) is negative.

We use the training data set of approximately 1700 MSAs described above to estimate a 400 × 400 doubly stochastic matrix* D*_CompMut_. This matrix is our mathematical model of how dissimilar compensatory mutations have affected genomic sequences in the course of evolution. Its training consists of five phases.

*Phase 1.* We calculate a signal and a null set of column pairs. The signal set consists of all (U,M)-significant column pairs, where* M* ranges over all training MSA. The null set consists of sufficiently many column pairs randomly chosen from every training MSA. For both the signal set and the null set we compute a symmetric 400 × 400 integer-valued matrix of frequencies of pair substitutions* C*_alt _and* C*_null_. To this end, the method used to compute BLOSUM62 matrices [53] is applied to count residue pair substitutions in MSA column pairs rather than residue substitution in columns.

*Phase 2.* Using* C*_alt _and* C*_null_, we define the matrix* C*_sig _by

$$\begin{array}{lll} & C_{\text{sig}}\left(\left(a_{i},a_{j}),(a_{k},a_{l}\right)\right)\; & \;\\ & :=\left\{\begin{array}{ll} C_{\text{alt}}\left(\left(a_{i},a_{j}),(a_{k},a_{l}\right)\right) & \text{if}\;\varphi \left(\left(a_{i},a_{j}),(a_{k},a_{l}\right)\right)=1;\\ 0 & \text{otherwise}; \end{array}\right)\; & \; \end{array}$$

where* φ*((*a*_*i*_*,a*_*j*_),(*a*_*k*_*,a*_*l*_)) = 1 if and only if (*a*_*i*_,*a*_*j*_) = (*a*_*k*_,*a*_*l*_) or

$$\begin{array}{lll} & \frac{C_{\text{alt}}\left(\left(a_{i},a_{j}),(a_{k},a_{l}\right)\right)}{\underset{i^{\prime },j^{\prime },k^{\prime },l^{\prime }}{\sum }C_{\text{alt}}\left(\left(a_{i^{\prime }},a_{j^{\prime }}),(a_{k^{\prime }},a_{l^{\prime }}\right)\right)}\; & \;\\ & >\frac{C_{\text{null}}\left(\left(a_{i},a_{j}),(a_{k},a_{l}\right)\right)}{\underset{i^{\prime },j^{\prime },k^{\prime },l^{\prime }}{\sum }C_{\text{null}}\left(\left(a_{i^{\prime }},a_{j^{\prime }}),(a_{k^{\prime }},a_{l^{\prime }}\right)\right)}.\; & \; \end{array}$$

*Phase 3.* We set all entries of the matrix* C*_sig_ outside the main diagonal that do not represent a formal dissimilar compensatory mutation to zero. This results in the matrix* C*_CompMut_. By normalizing* C*_CompMut_, we obtain a symmetric matrix* P*_CompMut_. For* a*_*i*_,*a*_*j*_,*a*_*k*_,*a*_*l*_ ranging over all amino acids,*P*_CompMut_((*a*_*i*_,*a*_*j*_),(*a*_*k*_,*a*_*l*_)) represents an empirical probability distribution on pairs of amino acid pairs.

*Phase 4.* We calculate the symmetric 400 × 400-matrix

$$\begin{array}{lll} & \;S_{\text{CompMut}}\;:=\;{\left(\;\log\frac{P_{\text{CompMut}}\;\left(\left(a_{i},a_{j}),(a_{k},a_{l}\right)\right)}{P_{\text{CompMut}}^{b}\;\left(a_{i},a_{j}\right)P_{\text{CompMut}}^{b}\;\left(a_{k},a_{l}\right)}\;\right)}_{\;\;(a_{i},a_{j}),(a_{k},a_{l})}\;,\; & \; \end{array}$$

where $\left(P_{\text{CompMut}}^{b}\left(a_{i},a_{j}\right)\right)$ is the marginal distribution of* P*_CompMut_.

*Phase 5.* We set all negative entries of* S*_CompMut_ to zero. Then we compute the doubly stochastic matrix* D*_CompMut _by means of the canonical iterated row-column normalization procedure [54].

Now we define our new $U_{D(\alpha )}$-metric based on* D*_CompMut_. For every column pair (*i*,*j*) of the input MSA* M*, we linearly transform the associated empirical pair distribution with the doubly stochastic matrix

$$D(\alpha ):=(1-\alpha )1+\alpha \;D_{\text{CompMut}}$$

where **1** is the 400 × 400 unit matrix,* D*_CompMut_ is the result of training phase 5, and* α *∈(0,1] is a preassigned real number. $U_{D(\alpha )}\left(i,j\right)$ is then defined to be the U-value (see Equation 1) of this transform.

Having canonically carried over the definition of a significant site pair and of the connectivity degree of a site to this case, a site* i* is called $\left(U_{D(\alpha )},M\right)$*-significant*, if* i*’s connectivity degree with respect to the metric $U_{D(\alpha )}$ is greater than or equal to the 90-th percentile.

Finally, a site is defined to be* CMF-significant* with respect to the MSA*M*, if it is (U,M)-significant or $\left(U_{D(\alpha )},M\right)$-significant. The*CMF*-significant sites are predicted as functionally or structurally important ones.

Principally, the controlling parameter* α *∈ (0,1] can be adjusted by the user. We set* α* to 1 to allow the two sets of (U,M)-significant and $\left(U_{D(\alpha )},M\right)$-significant positions to complement each other.

Note, that the matrix* S*_CompMut _could be replaced with another scoring matrix meaningful in this context.

## Competing interests

The authors declare that they have no competing interests.

## Authors’ contributions

SW developed the model underlying CMF. MG developed the model together with SW, designed and implemented the tool, and interpreted the results together with NT and MH. All authors read and approved the manuscript.

## Supplementary Material

Additional file 1 **EGFR significant sites. ***CMF*-significant residue sites of the human epidermal growth factor receptor (EGFR) protein. Click here for file

Additional file 2 **GCK significant sites. ***CMF*-significant residue sites of the human glucokinase (GCK) protein. Click here for file

Additional file 3 **Pdb entries of training MSAs.** Pdb entries of redundancy free data set. Click here for file
